# Ciprofloxacin prophylaxis in high risk neutropenic patients: effects on outcomes, antimicrobial therapy and resistance

**DOI:** 10.1186/1471-2334-13-356

**Published:** 2013-07-31

**Authors:** Marcia Garnica, Simone A Nouér, Flávia LPC Pellegrino, Beatriz M Moreira, Angelo Maiolino, Marcio Nucci

**Affiliations:** 1 Hospital Universitário Clementino Fraga Filho, Universidade Federal do Rio de Janeiro, Rua Professor Rodolpho Paulo Rocco 255, Cidade Universitária, Rio de Janeiro 21941-913, Brazil; 2 Instituto de Microbiologia Paulo de Góes, Universidade Federal do Rio de Janeiro, Rio de Janeiro, Brazil

**Keywords:** Neutropenia, Prophylaxis, Quinolone, Febrile neutropenia, Resistance

## Abstract

**Background:**

The use of quinolone prophylaxis in high-risk neutropenic patients is considered standard of care but the development of resistance is a concern. Previous studies have focused mainly on quinolone resistance among patients receiving prophylaxis, with very few data reporting its impact on the hospital microbial epidemiology.

**Methods:**

We analyzed a cohort of 329 episodes of chemotherapy-induced neutropenia in adults, and compared two periods: 2005 (period 1, no prophylaxis, n=110) and 2006-2008 (period 2, ciprofloxacin prophylaxis, n=219). Outcomes analyzed were the frequency of febrile neutropenia, bacteremia, duration of antibiotic therapy and hospitalization, and antimicrobial resistance to ciprofloxacin and extended-spectrum beta-lactamase [ESBL] production. We analyzed resistance rates (by patients-day) in the cohort, as well as in other patients (neutropenic and non-neutropenic, 11,975 patients-day) admitted to the hematology unit in the same period, taking into consideration the general resistance patterns in the hospital.

**Results:**

Quinolone prophylaxis (period 2) resulted in fewer episodes of febrile neutropenia (159/219 [73%] vs. 102/110 [93%], Chi-square 18.09, *p* = 0.00002), and bacteremia (49/219 [22] vs. 36/110 [33%], Chi-square 4.10, *p* = 0.04), shorter duration of antibiotic therapy (*p* = 0.0002) and hospitalization (*p* = 0.002), but more frequent use of carbapenems (79/219 [36%] vs. 15/110 [14%], Chi-square 18.06, *p* = 0.0002). In addition, period 2 was associated with higher rates of quinolone resistance (6.77 vs. 3.02 per 1,000 patients-day, *p* = 0.03). The rate of ESBL-producing enterobacteria in the two periods was slightly higher in patients receiving quinolone prophylaxis (1.27 vs. 0.38 per 1,000 patients-day, *p* = 0.26) as well as in the hematology unit overall (1.59 vs. 0.53 per 1,000 patients-day, *p* = 0.08), but remained stable in the whole hospital (0.53 vs. 0.56 per 1,000 patients-day, *p* = 0.74).

**Conclusions:**

Ciprofloxacin prophylaxis was beneficial in high risk neutropenic patients, but important modifications in the prescription of carbapenems and on antimicrobial resistance patterns of isolates were observed. The importance of hospital or ward ecology must be taken into account when deciding for quinolone prophylaxis in high-risk neutropenic patients.

## Background

The use of quinolone prophylaxis in neutropenic cancer patients has been associated with a reduction in the incidence of bacterial infections and mortality [1-4]. However, an increase in quinolone resistance [5-7], as well as resistance to other classes of antimicrobial agents, such as extended-spectrum beta-lactamase (ESBL) production among enterobacteria has been reported [8-12]. The potential increase in resistance with the use of quinolones is relevant in the neutropenic setting, because the high incidence of infection caused by ESBL-producing bacteria limits the options for empirical treatment of febrile neutropenia. Furthermore, in a setting of a high prevalence of Gram-negative resistance, the use of inappropriate antibiotic therapy results in significant excess mortality [13]. Studies reporting antimicrobial resistance among neutropenic patients receiving quinolone prophylaxis have focused mostly on the emergence of quinolone resistance among patients receiving prophylaxis, with very few data reporting its impact on the hospital microbial epidemiology [5,14].

In Brazil the rates of Gram-negative bacteremia in neutropenic patients are high [15], rendering quinolone prophylaxis an attractive approach. However, the rates of resistance among Gram-negative bacilli are high in the region [16]. Therefore, an analysis of the potential benefits of quinolone prophylaxis must be carefully weighed against the risks of resistance. In the present study we evaluated the impact of quinolone prophylaxis given during neutropenia on different outcomes, with special attention to the rates of resistance among patients on prophylaxis, as well as in patients admitted to the hematology unit in the same period, taking into consideration the general resistance patterns in the hospital.

## Methods

We conducted an observational study at Hospital Universitário Clementino Fraga Filho, Federal University of Rio de Janeiro, Brazil. This is a tertiary care hospital with ~400 beds, including a hematology and hematopoietic cell transplant (HCT) unit with 8 single-bed rooms with high efficiency particulate air (HEPA) filter and positive pressure, and five double-bed rooms without HEPA filter. The study was approved by the institution’s Ethical Committee (“Comitê de Ética em Pesquisa do Hospital Universitário Clementino Fraga Filho”).

Until 2006, no antibacterial prophylaxis had been given to neutropenic patients. Since then, all patients with hematological malignancies with an expected duration of neutropenia >7 days received prophylaxis with ciprofloxacin (500 mg orally twice a day, switched to 200 mg intravenously twice a day if the patient developed severe mucositis or intolerance to the oral formulation). Prophylaxis was started concomitantly with the induction chemotherapy or the conditioning regimen (HCT), and was maintained until bone marrow recovery or fever. In case of fever, blood cultures were obtained, and the patients were immediately started on intravenous cefepime, unless a previous episode of febrile neutropenia documented a cefepime-resistant Gram-negative organism. In this case, a carbapenem (imipenem or meropenem) was started. Blood cultures were repeated in case of persistent or recurrent fever, or as clinically indicated. Modifications in the empirical antibiotic regimen were performed according to the results of cultures and the clinical course of the patient.

For the purpose of this analysis, we selected all patients admitted between 2005 and 2008 who fulfilled the following criteria: a) receipt of chemotherapy with expected duration of neutropenia >7 days; and b) no fever or documentation of infection on the first day of chemotherapy. We then compared patients who received prophylaxis with ciprofloxacin (2006–2008, period 2, ciprofloxacin group) with patients who did not receive any antibiotic prophylaxis (2005, period 1, control group). Patients could be included more than once provided that more than 30 days had elapsed between two episodes of neutropenia. The groups were compared regarding demographic characteristics (age, gender), underlying disease, type of treatment (induction or intensification for acute leukemia), type of HCT (autologous or allogeneic), Karnofsky’s performance status, presence and severity of mucositis (Common Toxicity Criteria of National Cancer Institute World Health Organization criteria), and duration of neutropenia. We analyzed the following clinical outcomes: occurrence of fever, duration of empirical antibiotic therapy, bacteremia, bacteremia due to ciprofloxacin-resistant organism, bacteremia due to ESBL-producing enterobacteria, duration of hospitalization and antimicrobial therapy, use of carbapenem and glycopeptide, and death during the episode of neutropenia. All data were collected prospectively, as part of a large database of febrile neutropenia.

Neutropenia was defined as an absolute neutrophil count (ANC) < 500/mm^3^, and severe neutropenia as an ANC <100/mm^3^. Bone marrow recovery was defined as at least two consecutive ANCs >500/mm^3^, obtained on two separate days. Fever was defined as an axillary temperature >38°C. The febrile episodes were classified as fever of unknown origin (FUO), bacteremia, microbiologically documented infection without bacteremia, or clinically documented infection, as previously defined [17].

Polymicrobial bacteremia was defined if more than one pathogen was isolated from one or more blood cultures taken on the same day during the febrile episode. Blood cultures were processed with the BacT/ALERT system (Organon Teknika, USA). Bacterial identification and antimicrobial susceptibility tests were performed using the Vitek automated system (Bio-Merieux, Inc., France). No changes in microbiological procedures took place during the study period (2005-2008).

All bloodstream isolates from this cohort that had been stored (-80°C) were reprocessed to evaluate susceptibility. The following tests were performed: antimicrobial susceptibility test by disk-diffusion [18], minimal inhibitory concentration (MIC) of ciprofloxacin by E-test (Probac do Brasil), and determination of ESBL production by enterobacteria, with a double-diffusion test (using ceftazidime, cefepime, cefotaxime and aztreonam containing disks as substrates, and amoxicillin-clavulanate containing disks as inhibitor) [19]. If the isolate was not available for these additional procedures, data were obtained from the records of the microbiology laboratory.

In order to rule out the possibility that horizontal transmission of isolates occurred during the study period, molecular typing of Gram-negative isolates was performed using polymerase chain reaction (PCR)-fingerprinting with the following primers: ERIC-2 for *Escherichia coli*, *Klebsiella* spp., *Enterobacter* spp., and *Citrobacter freundii*[20,21], and 272 for *Serratia marcescens* and *Proteus mirabilis* isolates [22]. Banding patterns were interpreted by visual inspection and with GelCompar II (version 4.01), using the Dice index and the unweighted pair group method with arithmetic averages (UPGMA).

In addition to the analysis of resistance among patients in the cohort, we looked at the rates of resistance of bloodstream isolates obtained from patients of the unit who were not in the cohort, as well as the rates in other units of the hospital in the same 4-year period. Patients not in the cohort comprised 11,975 patients-day and included both neutropenic and non-neutropenic patients. We looked specifically at the rates of ciprofloxacin resistance and ESBL production. Resistance rates were reported per 1,000 patients.day.

All statistical analyses were performed using the SPSS for Windows software (version 11.0.1, SPSS, Inc., USA). The Chi-square test was used to compare proportions, and Mann–Whitney test to compare continuous variables; *p* values <0.05 were considered statistically significant.

## Results

We analyzed 220 patients (141 in the ciprofloxacin and 79 in the control group) who developed 329 episodes of neutropenia, 219 receiving ciprofloxacin prophylaxis (ciprofloxacin group) and 110 without quinolone prophylaxis (control group). Patients in the ciprofloxacin and control group had similar ages (mean 40 years, range 14 – 82 and 41 years, range 12 – 66, respectively, *p*=0.56) and gender (60% and 65% of males, respectively, *p* = 0.28). Ciprofloxacin prophylaxis was given for a mean of 11 days (range 1 – 30, SD ± 4.44), and was started before neutropenia in 94% of episodes, at a mean of 7 days before neutropenia (8 – 23, SD ± 3.49).

Table 1 shows the characteristics and outcomes of the episodes of neutropenia in the two groups. The duration of neutropenia was slightly shorter in the ciprofloxacin group (9 vs. 11 days, *p* = 0.02). In addition, mucositis (at any grade, but not grades 3 or 4) was less frequent in ciprofloxacin recipients (52% vs. 70%, *p* = 0.003). Febrile episodes were significantly less frequent in the ciprofloxacin group (73 vs. 93%, *p* < 0.001), and when present, occurred later in the course of neutropenia (median 4 vs. 2 days after the first day of neutropenia, *p* < 0.001). While the proportion of episodes classified as FUO and clinically documented infection was similar in the two groups, bacteremia was significantly less frequent in the ciprofloxacin group (22% vs. 33%, *p* = 0.04). In addition, the mean duration of hospitalization was shorter in the ciprofloxacin group (22 vs. 24 days, *p* = 0.002), as was the mean duration of antibiotic therapy (8 vs. 11 days, *p* < 0.001) (Additional file 1).

**Table 1:** Characteristics and outcomes of 329 neutropenic episodes in 220 patients who received or not ciprofloxacin prophylaxis during neutropenia

**Variable**	**Ciprofloxacin group N=219**	**Control group N=110**	***p *value**
Underlying disease			
Acute myeloid leukemia	48 (22)	22 (20)	0.69
Acute lymphoid leukemia	48 (22)	24 (22)	0.98
Multiple myeloma	56 (26)	31 (28)	0.61
Non-Hodgkin lymphoma	40 (18)	8 (7)	0.008
Hodgkin lymphoma	18 (8)	15 (14)	0.12
Other*	9 (4)	10 (9)	-
Autologous HCT	89 (41)	53 (48)	0.19
Allogeneic HCT	30 (14)	14 (13)	0.81
Central venous catheter	140 (64)	73 (66)	0.66
Performance status <50%	25 (11)	19 (17)	0.14
Mucositis, any grade	114 (52)	76 (70)	<0.001
Grade 3 or 4	28 (13)	16 (15)	0.66
Duration (days) of neutropenia, mean ±SD (range)	9 ± 6.3 (2–47)	11 ± 7.9 (2–61)	0.02
Duration (days) of severe neutropenia, mean ±SD (range)	7 ± 5.2 (1–38)	8 ± 6.9 (0–40)	0.39
Fever	159 (73)	102 (93)	<0.001
Fever of unknown origin	86 (39)	52 (47)	0.16
Bacteremia	49 (22)	36 (33)	0.04
due to a single Gram-negative	19 (9)	13 (12)	0.36
due to a single Gram-positive	22 (10)	18 (16)	0.10
Polymicrobial	8 (4)	5 (4.5)	0.77
Microbiologically documented without bacteremia	3 (1)	1 (1)	1.00
Clinically documented	21 (10)	12 (11)	0.71
Duration of hospitalization (days), mean ±SD (range)	22 ± 13.9 (4 – 97)	24 ± 10.4 (5 – 57)	0.002
Duration of antimicrobial treatment (days) , mean ±SD (range)	8 ± 7.6 (0 – 40)	11 ± 7.0 (0 – 33)	<0.001
Receipt of carbapenem**	79 (36)	15 (14)	<0.001
Receipt of glycopeptide	26 (24)	14 (13)	0.82
Death	20 (9)	12 (11)	0.61

Data are number (%) of episodes, unless otherwise indicated.

* Other underlying diseases: Ciprofloxacin group: aplastic anemia (5 episodes), and chronic myeloid leukemia and germ cell tumor (1 each); control group: chronic myeloid leukemia (9), and aplastic anemia (1).

** As empirical therapy in 43 episodes in the ciprofloxacin group and 1 in the control group; as a modification of the empirical regimen in 14 episodes in the ciprofloxacin group and in 36 episodes in the control group.

The rates of clinical or microbiological failure to the empirical treatment with cefepime were similar in the ciprofloxacin and control groups (12% vs. 8% for clinical failures, *p* = 0.33; and 8.5% and 6% for microbiological failures, *p* = 0.14; respectively). In addition, no differences were observed in the frequency of glycopeptide use between the two groups. However, carbapenems were given more frequently to ciprofloxacin recipients (36% vs. 14%, *p* < 0.001).

A total of 98 bacterial isolates were recovered from the 85 episodes of bacteremia. In the ciprofloxacin group, there were 19 episodes with documentation of a single Gram-negative, 22 episodes with a single Gram-positive, and 8 episodes of polymicrobial bacteremia, whereas in the control group there were 13 episodes with documentation of a single Gram-negative, 18 episodes of a single Gram-positive isolate, and 5 episodes of polymicrobial bacteremia (Table 1). Table 2 shows the species distribution of bloodstream isolates in both groups. The most frequent Gram-negative bacteria were *E. coli* and *P. aeruginosa*.

**Table 2:** Species distribution of 98 bacterial bloodstream isolates recovered from patients who received ciprofloxacin prophylaxis or not during neutropenia

**Microorganism**	**Ciprofloxacin group**	**Control group**
**Gram-positive**	N=28	N=24
Coagulase-negative staphylococci	14	16
*Staphylococcus aureus*	4	1
*α-* Hemolytic streptococci	5	4
Other Gram-positive*	5	3
**Gram-negative**	N=29	N=17
*Escherichia coli*	9	5
*Klebsiella pneumoniae*	2	1
Other enterobacteria**	4	4
*Pseudomonas aeruginosa*	3	4
Other non-fermentative Gram-negative***	11	3

The total number of isolates exceeds the number of bacteremias because there were 13 episodes of polymicrobial bacteremia.

* Ciprofloxacin group: *Enterococcus* sp. and *Streptococcus pneumoniae* (1 each), and 3 Gram-positive rods; Control group: *Enterococcus* sp. (2) and *Bacillus* sp. (1).

** Ciprofloxacin group: *Enterobacter* sp. (3), and *Shigella* sp. (1); Control group: *Citrobacter* sp. (2), and *Enterobacter* sp. and *Serratia* sp. (1 each).

*** Ciprofloxacin group: 5 *Acinetobacter* sp., 3 *Stenotrophomonas maltophilia*, and 3 *Burkholderia cepacia*; Control group: 1 *Acinetobacter* sp., 1 *Stenotrophomonas maltophilia*, and 1 *Burkholderia cepacia.*

Table 3 shows the resistance rates (per 1,000 patients.day) in the two periods. The rate of quinolone-resistant bacteremia (both Gram-positive and Gram-negative) was significantly higher in patients receiving quinolone prophylaxis (6.77 vs. 3.02 per 1,000 patients.day, *p* = 0.03). In addition, quinolone-resistant enterobacteria were more frequently isolated in period 2 both in cohort patients and in the hematology unit overall (2.12 vs. 0.38 per 1,000 patients.day, *p* = 0.06 in cohort patients, and 2.54 vs. 0.53 per 1,000 patients.day in the hematology unit, *p* = 0.004), but remained stable in the hospital (0.76 in period 1 and 0.64 in period 2, *p* = 0.15). The rates of ESBL production among enterobacteria increased slightly both in cohort patients (0.38 in period vs.1.27 in period 2, *p* = 0.26) and in the hematology unit (0.52 in period 1 vs. 1.59 in period 2, *p* = 0.08), and remained stable in the hospital (0.56 in period 1 vs. 0.53 in period 2, *p* = 0.74). No changes in quinolone resistance among staphylococci were observed in cohort patients.

**Table 3:** Incidence rates (per 1,000 patients-day) of resistant bacteria

	**Period 1 (2005)**	**Period 2 (2006–2008)**	***p *value**
**Cohort patients**			
Bacteremia due to Cip-R organisms*	3.02	6.77	0.03
Cip-R enterobacteria	0.38	2.12	0.06
Cip-R *Pseudomonas aeruginosa*	0.38	0.63	0.71
ESBL production	0.38	1.27	0.26
Cip-R *Staphylococcus aureus*	0	0.63	0.26
Cip-R CONS	2.27	2.75	0.72
**Non-cohort patients in the hematology unit**			
Cip-R enterobacteria	0.53	2.54	0.004
Cip-R *Pseudomonas aeruginosa*	0.70	0.32	0.39
ESBL production	0.52	1.59	0.08
Cip-R *Staphylococcus aureus*	0	0.16	0.52
Cip-R CONS	2.64	2.23	0.65
**Hospital**			
Cip-R enterobacteria	0.76	0.64	0.15
Cip-R *Pseudomonas aeruginosa*	0.22	0.21	0.78
ESBL production	0.56	0.53	0.74
Cip-R *Staphylococcus aureus*	0.33	0.23	0.06
Cip-R CONS	0.68	0.60	0.33

*Cip-R* = ciprofloxacin-resistant; *ESBL* = extended spectrum beta-lactamase; *CONS* = coagulase-negative staphylococci; *Total number of bacteremia due to quinolone-resistant organisms available in the cohort only.

**Figure 1:**
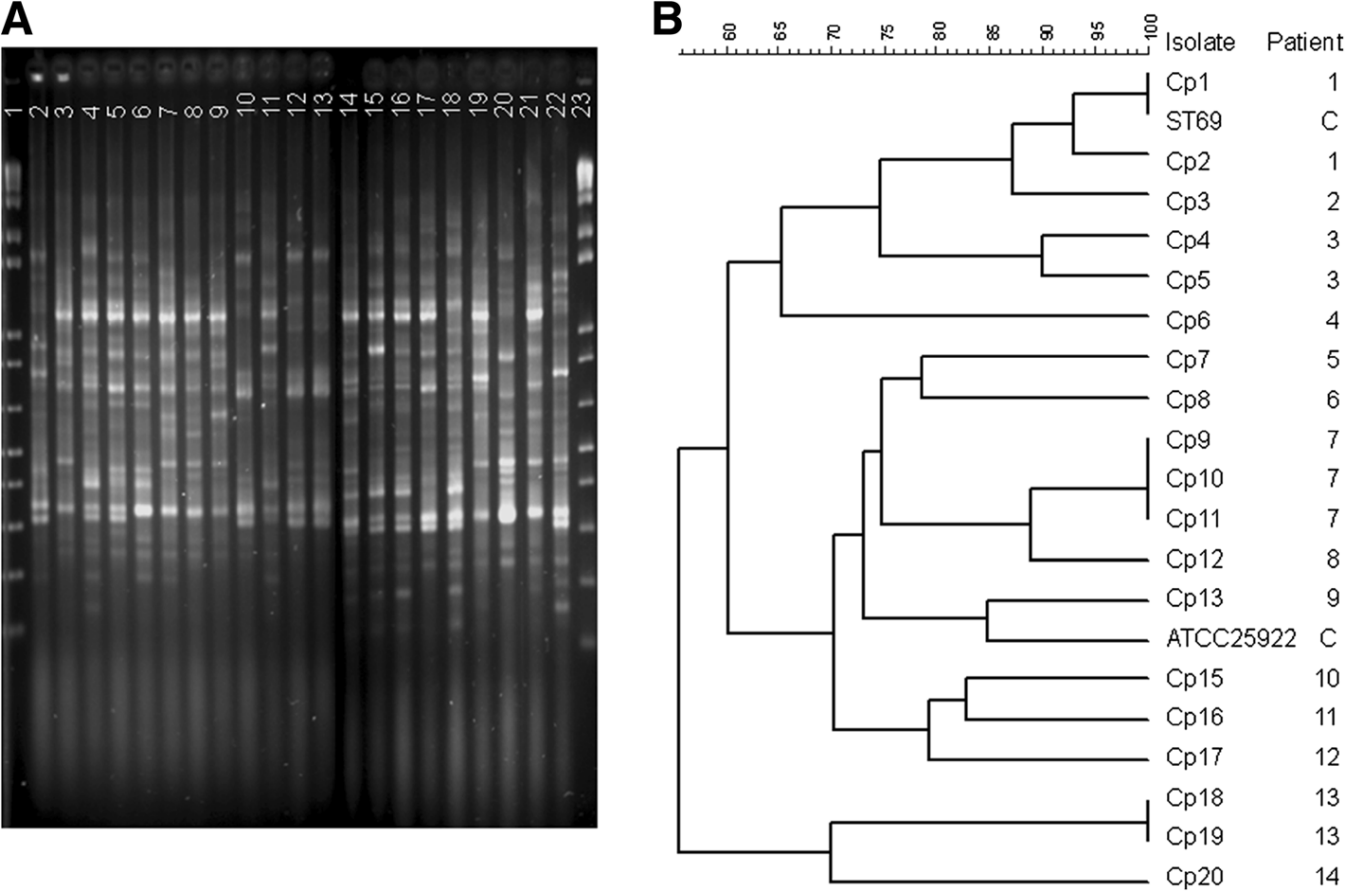
**Molecular analysis of Escherichia coli isolates from cohort patients. A.** ERIC2-PCR profiles of *Escherichia coli* isolates. Lanes 1 and 23: 1 Kb plus molecular size marker; Lanes 2–20: clinical isolates; Lanes 21 and 22: control strains ST69 and ATCC25922. **B.** Dendrogram of ERIC-2 PCR profiles of *Escherichia coli* isolates. C: control isolates.

The genotypic analysis of the isolates from the cohort patients showed a great genetic diversity among isolates recovered from episodes of the cohort, with no similarity between isolates (Figure 1).

## Discussion

In this study we observed that ciprofloxacin prophylaxis in high-risk neutropenic patients was associated with a decrease (and delay) in the occurrence of fever, bacteremia, and duration of antibiotic therapy and hospitalization. On the other hand, an increase in the frequency of quinolone resistance was observed, not only in patients receiving quinolones but in the whole hematology unit. In addition, the incidence of bacteremia due to ESBL-producing enterobacteria increased in the hematology unit, while in the hospital it remained stable.

Quinolones have been used as prophylaxis in neutropenic patients since the early 1990s. While its use has been associated with favorable outcomes, the drawback of resistance has been always a concern. Nevertheless, quinolone prophylaxis is considered standard of care in high risk neutropenic patients admitted to centers in which quinolone resistance is <30% [23,24].

The beneficial effects of quinolone prophylaxis observed in the present study were reported in the majority of randomized clinical trials. In one multicenter trial conducted in Italy, the use of levofloxacin in patients with expected duration of neutropenia >7 days resulted in a reduction in episodes of febrile neutropenia and bacteremia [1]. In another trial, the same benefits were observed, but there was an increase in the use of carbapenems [25], as observed in the present study.

While these studies give support to the use of quinolone prophylaxis in a select group of neutropenic patients, some important aspects regarding antimicrobial resistance have not been addressed. For example, the Italian trial showed that the rate of levofloxacin-resistant Gram-negative bloodstream isolates was 4-fold higher in levofloxacin recipients, but data regarding the frequency of resistance to other antimicrobial classes, such as beta-lactams, were not provided [1]. Likewise, other studies emphasized the emergence of quinolone resistance but provided few data regarding increasing of resistance to other antimicrobials [26-28]. The relationship between quinolone exposure and the emergency of ESBL production among enterobacteria is well documented, and is thought to be a result of the mutant window, a phenomenon in which a quinolone-resistant isolate is more likely to acquire other mechanisms of resistance [9].

The increased use of carbapenem in the second period of our study was mostly as primary antibiotic regimen for the first fever (43 episodes in the ciprofloxacin group vs. 1 in the control group). The increased use of carbapenems in the empirical regimen may have been due to a concern of clinicians when we started to experience an increase in the rate of bacteremia due to ESBL-producing enterobacteria. Although not significant, there was a slight increase in the recovery of ESBL-producing enterobacteria in period 2 both in the cohort group and in the hematology unit. However, a similar trend was not observed in the hospital in the same period. These data indicate that the increase in ESBL in the hematology unit was not a reflection of an increase in ESBL in the hospital, and suggest a relationship between quinolone use and ESBL. In support of this hypothesis are various studies reporting a close association between quinolone resistance and ESBL [9,29]. Furthermore, the lack of genetic similarity among the bloodstream isolates suggests that horizontal transmission was not the main factor responsible for the increase in the incidence of quinolone-resistant and ESBL-producing enterobacteria. Therefore, a careful judgment of the risks and benefits of quinolone resistance should be advanced.

Our study is limited by the fact that we used a historical control group. However, except for time (two different periods) and antibacterial prophylaxis policy (ciprofloxacin or not), no other intervention was carried out that could strongly influence the results. Specifically, no changes in antimicrobial use in the unit or in microbiological procedures occurred in the two periods. Another limitation of our study is that not all bacteria recovered from blood cultures in the period were available for further laboratory analysis.

## Conclusion

In conclusion, ciprofloxacin prophylaxis was beneficial in high risk neutropenic patients, but important modifications in the prescription of carbapenems and on antimicrobial resistance patterns of isolates were observed. The importance of hospital or ward ecology must be taken into account when deciding for quinolone prophylaxis in high-risk neutropenic patients.

## Abbreviations

ESBL: Extended-spectrum beta-lactamase; HCT: Hematopoietic cell transplantation; HEPA: High efficiency particulate air; ANC: Absolute neutrophil count; FUO: Fever of unknown origin; MIC: Minimum inhibitory concentration; PCR: Polymerase chain reaction.

## Competing interests

The authors declare that they have no competing interests.

## Authors’ contributions

MG designed the study, collected and analyzed data, and drafted the paper; SAN designed the study, analyzed data, contributed with intellectual input for the discussion and reviewed the final version of the manuscript; FLPCP processed the isolates and approved the final version of the manuscript; BMM contributed with intellectual input for the discussion and reviewed the final version of the manuscript; AM contributed with intellectual input for the discussion and reviewed the final version of the manuscript; MN designed the study, analyzed data, contributed with intellectual input for the discussion, and reviewed and approved the final version of the manuscript. All authors read and approved the final manuscript.

## Pre-publication history

The pre-publication history for this paper can be accessed here:

http://www.biomedcentral.com/1471-2334/13/356/prepub

## Supplementary Material

Additional file 1 Distribution of duration of neutropenia (A), severe neutropenia (B), hospitalization (C) and antimicrobial treatment (D) in Control and Ciprofloxacin Groups by Box-plots Graphs. Click here for file

## References

[B1] Bucaneve G Micozzi A Menichetti F Martino P Dionisi MS Martinelli G Levofloxacin to prevent bacterial infection in patients with cancer and neutropenia N Engl J Med 2005 353 977 987 10.1056/NEJMoa044097 16148283

[B2] Cullen M Steven N Billingham L Gaunt C Hastings M Simmonds P Antibacterial prophylaxis after chemotherapy for solid tumors and lymphomas N Engl J Med 2005 353 988 998 10.1056/NEJMoa050078 16148284

[B3] Gafter-Gvili A Fraser A Paul M Leibovici L Meta-analysis: antibiotic prophylaxis reduces mortality in neutropenic patients Ann Intern Med 2005 142 979 995 10.7326/0003-4819-142-12_Part_1-200506210-00008 15968013

[B4] Reuter S Kern WV Sigge A Dohner H Marre R Kern P Impact of fluoroquinolone prophylaxis on reduced infection-related mortality among patients with neutropenia and hematologic malignancies Clin Infect Dis 2005 40 1087 1093 10.1086/428732 15791505

[B5] Gafter-Gvili A Paul M Fraser A Leibovici L Effect of quinolone prophylaxis in afebrile neutropenic patients on microbial resistance: systematic review and meta-analysis J Antimicrob Chemother 2007 59 5 22 17077101 10.1093/jac/dkl425

[B6] Leibovici L Paul M Cullen M Bucaneve G Gafter-Gvili A Fraser A Antibiotic prophylaxis in neutropenic patients: new evidence, practical decisions Cancer 2006 107 1743 1751 10.1002/cncr.22205 16977651

[B7] Von BH Sigge A Bommer M Kern WV Marre R Dohner H J Antimicrob Chemother 2006 58 891 894 10.1093/jac/dkl320 16880172

[B8] Kern WV Steib-Bauert M De WK Reuter S Bertz H Frank U Fluoroquinolone consumption and resistance in haematology-oncology patients: ecological analysis in two university hospitals 1999–2002 J Antimicrob Chemother 2005 55 57 60 15574472 10.1093/jac/dkh510

[B9] Paterson DL Mulazimoglu L Casellas JM Ko WC Goossens H Von GA Epidemiology of ciprofloxacin resistance and its relationship to extended-spectrum beta-lactamase production in Klebsiella pneumoniae isolates causing bacteremia Clin Infect Dis 2000 30 473 478 10.1086/313719 10722430

[B10] Schwaber MJ Navon-Venezia S Schwartz D Carmeli Y High levels of antimicrobial coresistance among extended-spectrum-beta-lactamase-producing Enterobacteriaceae Antimicrob Agents Chemother 2005 49 2137 2139 10.1128/AAC.49.5.2137-2139.2005 15855548 PMC1087677

[B11] Spanu T Luzzaro F Perilli M Amicosante G Toniolo A Fadda G Occurrence of extended-spectrum beta-lactamases in members of the family Enterobacteriaceae in Italy: implications for resistance to beta-lactams and other antimicrobial drugs Antimicrob Agents Chemother 2002 46 196 202 10.1128/AAC.46.1.196-202.2002 11751134 PMC126983

[B12] Lautenbach E Strom BL Bilker WB Patel JB Edelstein PH Fishman NO Epidemiological investigation of fluoroquinolone resistance in infections due to extended-spectrum beta-lactamase-producing Escherichia coli and Klebsiella pneumoniae Clin Infect Dis 2001 33 1288 1294 10.1086/322667 11565067

[B13] Trecarichi EM Tumbarello M Spanu T Caira M Fianchi L Chiusolo P Incidence and clinical impact of extended-spectrum-beta-lactamase (ESBL) production and fluoroquinolone resistance in bloodstream infections caused by Escherichia coli in patients with hematological malignancies J Infect 2009 58 299 307 10.1016/j.jinf.2009.02.002 19272650

[B14] Wener KM Schechner V Gold HS Wright SB Carmeli Y Treatment with fluoroquinolones or with beta-lactam-beta-lactamase inhibitor combinations is a risk factor for isolation of extended-spectrum-beta-lactamase-producing Klebsiella species in hospitalized patients Antimicrob Agents Chemother 2010 54 2010 2016 10.1128/AAC.01131-09 20211888 PMC2863615

[B15] Oliveira AL De SM Carvalho-Dias VM Ruiz MA Silla L Tanaka PY Epidemiology of bacteremia and factors associated with multi-drug-resistant gram-negative bacteremia in hematopoietic stem cell transplant recipients Bone Marrow Transplant 2007 39 775 781 10.1038/sj.bmt.1705677 17438585

[B16] Gales AC Castanheira M Jones RN Sader HS Antimicrobial resistance among Gram-negative bacilli isolated from Latin America: results from SENTRY Antimicrobial Surveillance Program (Latin America, 2008–2010) Diagn Microbiol Infect Dis 2012 73 354 360 10.1016/j.diagmicrobio.2012.04.007 22656912

[B17] From the Immunocompromised Host Society The design, analysis, and reporting of clinical trials on the empirical antibiotic management of the neutropenic patient. Report of a consensus panel J Infect Dis 1990 161 397 401 10.1093/infdis/161.3.397 2179421

[B18] Performance Standards for Antimicrobial Susceptibility Testing M100 S20 2010 USA: Clinical and Laboratory Standards Institute 10.1128/JCM.00213-21 PMC8601225 34550809

[B19] Jarlier V Nicolas MH Fournier G Philippon A Extended broad-spectrum beta-lactamases conferring transferable resistance to newer beta-lactam agents in Enterobacteriaceae: hospital prevalence and susceptibility patterns Rev Infect Dis 1988 10 867 878 10.1093/clinids/10.4.867 3263690

[B20] Renders N Romling Y Verbrugh H Van BA Comparative typing of Pseudomonas aeruginosa by random amplification of polymorphic DNA or pulsed-field gel electrophoresis of DNA macrorestriction fragments J Clin Microbiol 1996 34 3190 3195 8940470 10.1128/jcm.34.12.3190-3195.1996 PMC229481

[B21] Versalovic J Koeuth T Lupski JR Distribution of repetitive DNA sequences in eubacteria and application to fingerprinting of bacterial genomes Nucleic Acids Res 1991 19 6823 6831 10.1093/nar/19.24.6823 1762913 PMC329316

[B22] Mahenthiralingam E Campbell ME Foster J Lam JS Speert DP Random amplified polymorphic DNA typing of Pseudomonas aeruginosa isolates recovered from patients with cystic fibrosis J Clin Microbiol 1996 34 1129 1135 8727889 10.1128/jcm.34.5.1129-1135.1996 PMC228968

[B23] Freifeld AG Bow EJ Sepkowitz KA Boeckh MJ Ito JI Mullen CA Clinical practice guideline for the use of antimicrobial agents in neutropenic patients with cancer: 2010 Update by the Infectious Diseases Society of America Clin Infect Dis 2011 52 427 431 10.1093/cid/ciq147 21205990

[B24] Wingard JR Eldjerou L Leather H Use of antibacterial prophylaxis in patients with chemotherapy-induced neutropenia Curr Opin Hematol 2012 19 21 26 10.1097/MOH.0b013e32834da9bf 22080847

[B25] Eleutherakis-Papaiakovou E Kostis E Migkou M Christoulas D Terpos E Gavriatopoulou M Prophylactic antibiotics for the prevention of neutropenic fever in patients undergoing autologous stem-cell transplantation: results of a single institution, randomized phase 2 trial Am J Hematol 2010 85 863 867 10.1002/ajh.21855 20882526

[B26] Chong Y Yakushiji H Ito Y Kamimura T Clinical impact of fluoroquinolone prophylaxis in neutropenic patients with hematological malignancies Int J Infect Dis 2011 15 e277 e281 10.1016/j.ijid.2010.12.010 21324723

[B27] Craig M Cumpston AD Hobbs GR Devetten MP Sarwari AR Ericson SG The clinical impact of antibacterial prophylaxis and cycling antibiotics for febrile neutropenia in a hematological malignancy and transplantation unit Bone Marrow Transplant 2007 39 477 482 10.1038/sj.bmt.1705591 17322937

[B28] Gomez L Garau J Estrada C Marquez M Dalmau D Xercavins M Ciprofloxacin prophylaxis in patients with acute leukemia and granulocytopenia in an area with a high prevalence of ciprofloxacin-resistant Escherichia coli Cancer 2003 97 419 424 10.1002/cncr.11044 12518366

[B29] Cremet L Caroff N Dauvergne S Reynaud A Lepelletier D Corvec S Prevalence of plasmid-mediated quinolone resistance determinants in ESBL Enterobacteriaceae clinical isolates over a 1-year period in a French hospital Pathol Biol (Paris) 2011 59 151 156 10.1016/j.patbio.2009.04.003 19481883

