# Supplementary material for: Ciprofloxacin prophylaxis in high risk neutropenic patients: effects on outcomes, antimicrobial therapy and resistance
Source: BMC Infect Dis. 2013 Jul 31;13:356. doi: 10.1186/1471-2334-13-356 (PMC3729823; doi:10.1186/1471-2334-13-356)
Supplement: Additional file 1 — Distribution of duration of neutropenia (A), severe neutropenia (B), hospitalization (C) and antimicrobial treatment (D) in Control and Ciprofloxacin Groups by Box-plots Graphs. [file 1471-2334-13-356-S1.docx]

Distribution of duration of neutropenia (A), severe neutropenia (B), hospitalization (C) and antimicrobial treatment (D) in Control and Ciprofloxacin Groups by Box-plots Graphs


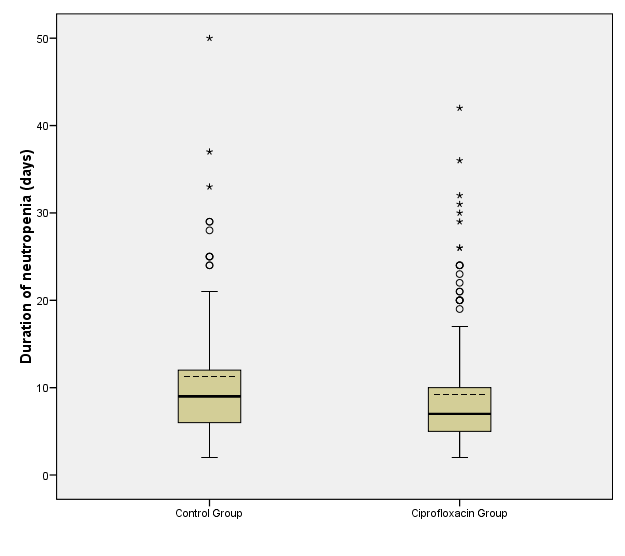

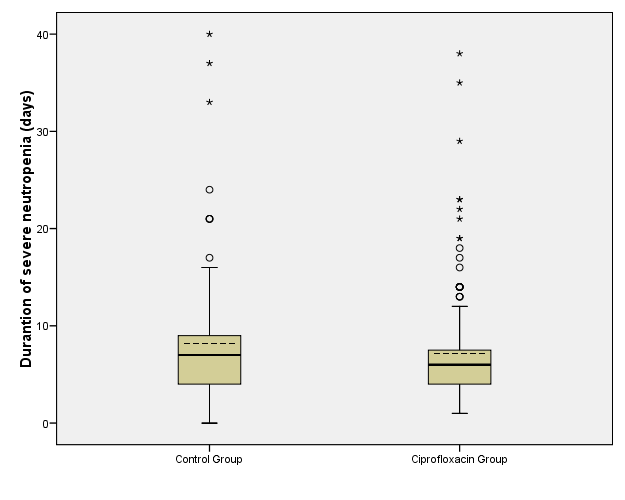


A

B

Median

Mean

Median

Mean


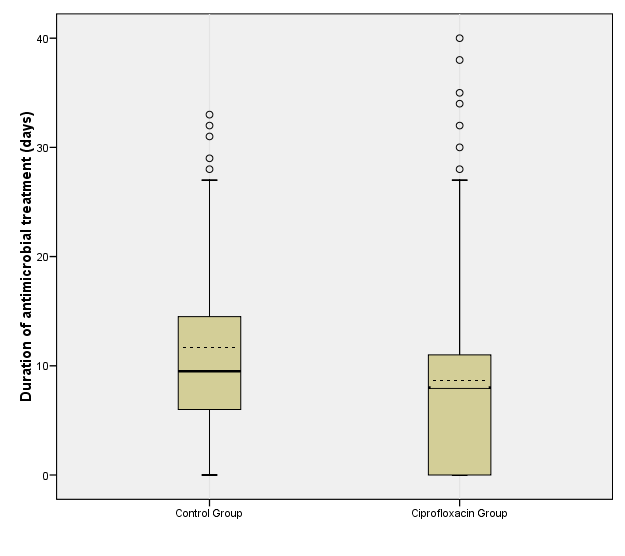

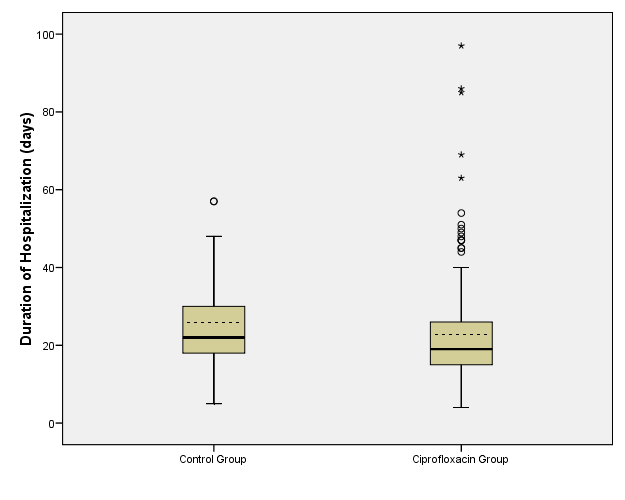


D

C

Median

Mean

Median

Mean
